# Supplementary material for: Determinants of non-adherence to treatment for tuberculosis in high-income and middle-income settings: a systematic review protocol
Source: BMJ Open. 2018 Jan 21;8(1):e019287. doi: 10.1136/bmjopen-2017-019287 (PMC5781023; doi:10.1136/bmjopen-2017-019287)
Supplement: Supplementary file 1 [file bmjopen-2017-019287supp001.pdf]

Determinants of non-adherence to treatment for tuberculosis in high- and middle-income settings: a systematic review protocol

Search strategy for Medline

1. (tuberculosis.mp. or exp tuberculosis, human/ or TB/) not latent.mp. [mp=title, abstract, original title, name of substance word, subject heading word, keyword heading word, protocol supplementary concept word, rare disease supplementary concept word, unique identifier, synonyms]

2. exp patient acceptance of health care/

3. adher*.mp.

4. ((non or "not") adj3 initiat*).mp.

5. ((non or "not") adj3 complet*).mp.

6. ((non or "not") adj3 complian*).mp.

7. lost to follow up.mp.

8. nonadher*.mp.

9. ((non or "not") adj3 concord*).mp.

10. (DOTS or directly observed therap* or treat*).mp.

11. 2 or 3 or 4 or 5 or 6 or 7 or 8 or 9 or 10

12. 1 and 11
