# Supplementary material for: Determinants of non-adherence to treatment for tuberculosis in high-income and middle-income settings: a systematic review protocol
Source: BMJ Open. 2018 Jan 21;8(1):e019287. doi: 10.1136/bmjopen-2017-019287 (PMC5781023; doi:10.1136/bmjopen-2017-019287)
Supplement: Author's manuscript [file bmjopen-2017-019287.draft_revisions.pdf]

BMJ Open

BMJ Open is committed to open peer review. As part of this commitment we make the peer review history of every article we publish publicly available.

When an article is published we post the peer reviewers' comments and the authors' responses online. We also post the versions of the paper that were used during peer review. These are the versions that the peer review comments apply to.

The versions of the paper that follow are the versions that were submitted during the peer review process. They are not the versions of record or the final published versions. They should not be cited or distributed as the published version of this manuscript.

BMJ Open is an open access journal and the full, final, typeset and author-corrected version of record of the manuscript is available on our site with no access controls, subscription charges or pay-per-view fees (http://bmjopen.bmj.com).

If you have any questions on BMJ Open's open peer review process please email editorial.bmjopen@bmj.com

BMJ Open

Determinants of non-adherence to treatment for tuberculosis in high- and middle-income settings: a systematic review protocol

Journal:	*BMJ Open*
Manuscript ID	bmjopen-2017-019287
Article Type:	Protocol
Date Submitted by the Author:	15-Sep-2017
Complete List of Authors:	Wurie, Fatima; University College London, Institute of Health Informatics; University College London, School of Pharmacy<br>Cooper, Vanessa; University College London, UCL School of Pharmacy<br>Horne, Robert; University College London, UCL School of Pharmacy<br>Hayward, Andrew; University College London, UCL Institute of Epidemiology and Health Care; University College London, UCL Institute of Health Informatics
**Primary Subject Heading**:	Infectious diseases
Secondary Subject Heading:	Research methods, Public health
Keywords:	Tuberculosis < INFECTIOUS DISEASES, systematic review, treatment adherence

SCHOLARONE™
Manuscripts

only

Determinants of non-adherence to treatment for tuberculosis in high- and middle-income settings: a systematic review protocol

Fatima B. Wurie MSc*[1,2], Vanessa Cooper PhD[2], Robert Horne PhD[2], Andrew C. Hayward MD[1,3]

[1]Institute of Health Informatics, University College London, UK

[2]Centre for Behavioural Medicine, School of Pharmacy, University College London, UK

[3]Institute of Epidemiology and Health Care, University College London, UK

***Corresponding author email address:** f.wurie@ucl.ac.uk

For peer review only

For peer review only - http://bmjopen.bmj.com/site/about/guidelines.xhtml

Abstract

Introduction Treatment for tuberculosis is highly effective if taken according to prescribed schedules. However, many people have difficulty adhering to treatment which can lead to poorer clinical outcomes, the development of drug resistance, increased duration of infectivity and consequent onward transmission of infection. A range of approaches are available to support adherence but in order to target these effectively a better understanding of the predictors of poor adherence is needed. This review aims to highlight the personal, socio-cultural and structural factors that may lead to poor adherence in high- and middle-income settings.

Methods and analysis Six electronic databases: Medline, EMBASE, CINAHL, PsychInfo, The Cochrane Library, Scopus and Web of Science will be searched for relevant articles using a pre-specified search strategy. Observational studies will be targeted to explore factors that influence adherence to treatment in individuals diagnosed with tuberculosis. Screening title and abstract followed by full-text screening and critical appraisal will be conducted by two researchers. Data will be extracted using the Population, Exposure, Comparator, Outcomes, Study characteristics (PECOS) framework. To assess study quality, we will use the Grading of Recommendations, Assessment, Development and Evaluation (GRADE) approach, in which quality of evidence is examined for each outcome rather than by individual study. A narrative synthesis of the studies will be compiled. A meta-analysis will be considered if there are sufficient numbers of studies that are homogenous in study design, population and outcomes.

Dissemination A draft conceptual framework will be identified that a) identifies key barriers to adherence at each contextual level (e.g. personal, socio-cultural, health systems) and b) maps the relationships, pathways, and mechanisms of effect between these factors and adherence outcomes for people with TB. The draft conceptual framework will guide targeting of adherence interventions and further research.

PROSPERO registration number: CRD42017061049

[289 words]

For peer review only

For peer review only - http://bmjopen.bmj.com/site/about/guidelines.xhtml

Background

Rationale

Internationally, adherence to treatment for tuberculosis is recognised as a key tenet of the TB Elimination Framework within low-incidence countries[i] yet little is known of the personal, social and cultural factors that drive non-adherence across different groups in these settings. Poor adherence is cited as the primary reason for sub-optimal clinical benefit[ii] and leads to poorer clinical outcomes, the development of drug resistance, increased duration of infectivity and consequent onward transmission of infection. DOTS (directly-observed therapy, short-course) is the international standard for TB control. The standard 'short-course' regimen to treat drug-sensitive TB is six months. For those diagnosed with multi-drug resistant TB (MDR-TB) this regimen increases to nine to 20 months and adverse effects are more common under these regimens. Despite the plethora of effective TB regimens that exist the treatment completion rates are low and vary across different groups and may hamper international efforts to control TB. The long duration of any current effective treatment regimen for TB can make it difficult for patients to take their drugs as prescribed. For example, in 2014 in England 73.3% of short-course treated patients completed treatment under these circumstances by six to eight months and 84.5% within a year[iii]. The outcome is worse for MDR or rifampicin resistant TB cases notified in 2013, where only 57.8% had done so by 24 months. These suggest that the effectiveness of TB treatment is considerably poor.

There are a range of approaches to support adherence to treatment for TB. These include self-reporting through attendance at follow-up appointments, collecting prescriptions from clinics, pill counts and pharmacy reports, electronic devices (medication events monitoring systems (MEMS) caps), urine inspection, testing for drug levels and directly-observed therapy (DOT) attendance or video-observed therapy (VOT) sessions[iv v] and the use of incentives and enablers to support adherence and engagement with services[vi].

Some studies have used quantitative methods to evaluate these approaches for TB, many of which been conducted in resource-poor settings. Other qualitative studies[vii] in resource-poor settings have highlighted that poverty and gender discrimination, the social context, health service factors and personal factors interact affecting adherence to treatment.

For peer review only - http://bmjopen.bmj.com/site/about/guidelines.xhtml

The NICE Medicines Adherence Guidelines[viii] recommended that support should be tailored to meet the needs of the individual by addressing both the perceptual factors (e.g. beliefs about the illness and treatment)[ix] and practical factors (e.g. capability, resources and opportunity) influencing the motivation and ability to start and continue with treatment[x]. This can be summarised as a Perceptions and Practicalities Approach (PAPA)[xi]. Research conducted in patients with a variety of long-term chronic conditions suggests that key beliefs influencing patients' common-sense evaluations of prescribed medicines have allowed the development of the Necessity-Concerns Framework (NCF)[xii] The extent to which these factors exist or are important in predicting non-adherence in resource-rich settings and narratives on perceptions of tuberculosis and its treatment regimens in the context of TB are yet to be explored.

Objectives

This systematic review aims to identify the personal, socio-cultural and structural factors associated with poor adherence to treatment for TB in high- and middle-income settings. A better understanding of these factors will better inform development of interventions to strengthen a patient-centred approach for the delivery of TB programmes and services.

Review questions:

What are the determinants of non-adherence to treatment in patients with tuberculosis in high- and middle-income settings?

For peer review only - http://bmjopen.bmj.com/site/about/guidelines.xhtml

Methods

Eligibility criteria

Study design/characteristics:

Inclusion criteria

Empirical studies employing prospective, longitudinal, cross-sectional or retrospective designs and quantitative or qualitative methods to explore barriers and facilitators to treatment uptake, adherence, continuation, completion for tuberculosis. Randomised and non-randomised prospective comparative studies of interventions will be included if any predictors were found to have increased uptake, adherence and continuation of TB treatment.

The active TB condition has to be defined in the study using a clinical diagnosis. For studies presenting treatment completion rates, a definition for completion will have to be provided.

Studies conducted in high- (a GNI per capita of $12,476 or more) and upper-middle-income settings (a GNI per capita between $4,036 and $12,475 as calculated using the World Bank Atlas method for the current 2017 fiscal year will be included in the review.

Exclusion criteria

Studies conducted in resource-limited settings (GNI per capita of $1,025 or less in 2015 as calculated by the World Bank Atlas methods for the current 2017 fiscal year will be excluded because reviews in this area have focused already on low- and low to middle-income settings[xiii]

No restrictions on age, gender or ethnicity of participants.

Participants: Individuals clinically diagnosed with active TB

Exposure: The primary exposures of interest are the risk factors that may influence adherence. Thus, studies reporting on patient demographics, knowledge and attitudes, characteristics of TB disease, social characteristics of patients, service-related factors and comorbidities will be included in the review.

Outcomes: Studies will be included in the review if the primary outcome is non-adherence. Non-adherence will be determined by self-reporting through attendance at follow-up appointments, collecting prescriptions

For peer review only - http://bmjopen.bmj.com/site/about/guidelines.xhtml

from clinics, pill counts and pharmacy reports, electronic devices (medication events monitoring systems (MEMS) caps), urine inspection, testing for drug levels and directly-observed therapy (DOT) attendance or video-observed therapy (VOT) sessions. Studies that report outcomes such as non-completion of treatment and/or lost to follow-up and/or treatment refusal and/or relapse of tuberculosis will also be included.

Information sources: Electronic databases: Medline, EMBASE, CINAHL, PsychInfo, The Cochrane Library, Scopus and Web of Science will be searched. The reference lists of relevant systematic reviews will be screened to find primary articles.

Search strategy: We will carry out medical subject heading (MeSH) terms and keyword searches for tuberculosis, treatment uptake, adherence, compliance and concordance. Our draft search strategy will combine MeSH and free text terms (including term explosion) for tuberculosis. No filters for study type will be applied for TB studies. We will remove editorials, news items and letters.

The list of proposed search terms will be reviewed by all authors and any necessary adjustments made prior to running the search. We will review the reference lists of eligible articles and relevant reviews to identify additional papers not indexed in the databases searched.

Data management: Output from the searched databases will be exported into Endnote version. X.7.7.1. and duplicate records removed electronically. Screening and extraction will occur in a Microsoft Access database to ensure that all retrieved references are fully tracked

Selection process: Articles will be selected by screening the title and abstract to assess whether they fulfil the study eligibility criteria. Two researchers (FW and VC) will conduct abstract selection and critical appraisal of the full-text articles. To reduce the risk of missing potentially relevant studies, researchers will adopt a more lenient approach at the first level of screening. Both researchers will obtain full-text articles for studies that meet the review inclusion criteria. Reasons for rejection of articles during the full-text screening process will be noted and any discrepancies will be discussed by FW and VC and consultation with AH and RH will be done if necessary.

Data extraction: We will use the Population, Exposure, Comparator, Outcomes, Study characteristics (PECOS) framework to systematise data extraction. Data will be extracted using a standardised template containing information on each of the following 5 domains:

For peer review only - http://bmjopen.bmj.com/site/about/guidelines.xhtml

Population: characteristics of the study population (e.g. sex and age distribution, ethnicity, immune status), recruitment and sampling methods, inclusion/exclusion criteria);

Exposure: any risk factors that may influence adherence. Including patient demographics, knowledge and attitudes of TB, characteristics of TB , social characteristics of patients, service-related factors, interventions and comorbidities, number of exposed subjects, any exclusions;

Comparators: identification and definition of unexposed subjects, any exclusions;

Outcomes: definition and identification of adherence levels for TB, non-completion of concomitant treatment, loss to follow-up, treatment refusal, TB relapse, number of subjects, any exclusions, length of follow-up;

Study characteristics: authors, publication year, setting/source of participants, design, period of study, length if follow-up time (if relevant), aims and objectives. Unadjusted and fully-adjusted effect estimates for the association between TB and adherence will be recorded. Details of confounders measured and adjusted will also be noted. Any results from additional stratified analyses will also be recorded. We will use a thematic analysis to synthesise findings from the qualitative studies.

We will consider contacting corresponding authors to obtain any missing information using a standardised email template.

Risk of bias quality assessment (in individual studies): We will use the Grading of Recommendations, Assessment, Development and Evaluation (GRADE)[xiv] approach, in which quality of evidence is examined for each outcome rather than by individual study. We will examine each outcome for risks of bias, inconsistency, indirectness, imprecision, publication bias and any additional domains deemed appropriate to categorise the strength of evidence as ‘high’, ‘moderate’, ‘low’ or ‘very low’. We will also consider expanding the risk of bias criteria to ensure full assessment of all domains relevant to observational studies such as participant selection, variable measurement and control for confounding, in line with the Cochrane Collaboration tool.

Strategy for data synthesis: A narrative synthesis will be compiled of the studies, including a consideration of the socio-economic context in which included interventions were implemented and other critical factors, such as the drug resistance profiles of the study population. The evidence tables will be arranged and divided according to the different treatment durations and regimens for treatment for TB.

For peer review only - http://bmjopen.bmj.com/site/about/guidelines.xhtml

For peer review only

Meta-analysis: If there are sufficient numbers of studies that are homogenous in study design, population and outcomes we will obtain a pooled effect estimate. The choice of fixed or random effects model will be guided by the level of statistical heterogeneity (assessed using the I^2 statistic).

Ethics and dissemination: Ethical review is not required as this study is a systematic review. It is our intention to submit the findings of this review to a peer-reviewed journal and to present at national and international symposia. Based on the results of the systematic review, we will develop a draft conceptual framework that a) identifies key barriers to adherence at each contextual level (e.g. personal, socio-cultural, health systems) and b) maps the relationships, pathways, and mechanisms of effect between these factors and adherence outcomes for TB patients on treatment. The draft conceptual framework will guide research questions and formative primary research to understand the factors that influence patterns of irregular and non-adherence.

[This protocol has been prepared using the Preferred Reporting Items for Systematic Reviews and meta-Analyses Protocols (PRISMA-P) guidelines[xv]]

Contributorship statement

ACH and FW conceived the idea, planned and designed the study protocol. FW planned the statitical analysis and data extraction. RH and VC provided critical insights. All authors have approved and contributed to the final written manuscript.

Competing interests

None

Funding statement

No funding to report for this submission

Data sharing statement

This study is a systematic review protocol and no unpublished data has arisen from this proposed work.

For peer review only - http://bmjopen.bmj.com/site/about/guidelines.xhtml

References

[i] World Health Organisation. TB Elimination Framework. Geneva. 2014
[ii] World Health Organisation. Adherence to long term therapies. Geneva; Jan. 2003 http://www.who.int/chp/knowledge/publications/adherence_report/en/
[iii] Public Health England. Tuberculosis in England: 2016. Sept. 2016. https://www.gov.uk/government/publications/tuberculosis-in-england-annual-report
[iv] Krueger et al. Videophone utilization as an alternative to directly observed therapy for tuberculosis. 2010 INT J TUBERC LUNG DIS 14(6):779–781
[v] Story A, Garfein RS, Hayward A, Rusovich V, Dadu A, Soltan V, et al. Monitoring Therapy Adherence of Tuberculosis Patients by using Video-Enabled Electronic Devices. Emerg Infect Dis. 2016;22(3):538-540. https://dx.doi.org/10.3201/eid2203.151620
[vi]Lutge EE, Wiysonge CS, Knight SE, Sinclair D, Volmink J. Incentives and enablers to improve adherence in tuberculosis. Cochrane Database of Systematic Reviews 2015, Issue 9. Art. No.: CD007952. DOI: 10.1002/14651858.CD007952.pub3

[viii] National Institute for Health and Clinical Excellence. Medicines adherence: involving patients in decisions about prescribed medicines and supporting adherence. NICE guidelines [CG76]. Jan 2009.
[ix] Horne R, Weinman J, Barber N, Elliott R, Morgan M. Concordance, adherence and compliance in medicine taking. Dec 2005. http://www.nets.nihr.ac.uk/__data/assets/pdf_file/0009/64494/FR-08-1412-076.pdf
[x] Chang S-H, Cataldo JK. A systematic review of global cultural variations in knowledge, attitudes and health responses to tuberculosis stigma. Int J Tuberc Lung Dis Off J Int Union Tuberc Lung Dis. 2014;18:168–73.
[xi] Horne R, Weinman J, Barber N, Elliott R, Morgan M. Concordance, adherence and compliance in medicine taking. Dec 2005. http://www.nets.nihr.ac.uk/__data/assets/pdf_file/0009/64494/FR-08-1412-076.pdf

[xii] Horne R, Chapman SCE, Parham R, Freemantle N, Forbes A, Cooper V (2013) Understanding Patients' Adherence-Related Beliefs about Medicines Prescribed for Long-Term Conditions: A Meta-Analytic Review of the Necessity-Concerns Framework. PLoS ONE 8(12): e80633. https://doi.org/10.1371/journal.pone.0080633
[xiii] Munro SA, Lewin SA, Smith HJ, Engel ME, Fretheim A, Volmink J. Patient Adherence to Tuberculosis Treatment: A Systematic Review of Qualitative Research. PLoS Med. 2007 Jul 24;4(7):e238
[xiv] Handbook for grading the quality of evidence and the strength of recommendations using the GRADE approach. Cochrane Collaboration. Updated October 2013.
[xv]Shamseer L, Moher D, Clarke M, et al. Preferred reporting items for systematic review and meta-analysis protocols (PRISMA-P) 2015: elaboration and explanation. BMJ 2015;349:g7647. doi:10.1136/bmj.g7647

For peer review only

For peer review only - http://bmjopen.bmj.com/site/about/guidelines.xhtml

BMJ Open

Determinants of non-adherence to treatment for tuberculosis in high- and middle-income settings: a systematic review protocol

Journal:	*BMJ Open*
Manuscript ID	bmjopen-2017-019287.R1
Article Type:	Protocol
Date Submitted by the Author:	16-Nov-2017
Complete List of Authors:	Wurie, Fatima; University College London, Institute of Health Informatics; University College London, School of Pharmacy<br>Cooper, Vanessa; University College London, UCL School of Pharmacy<br>Horne, Robert; University College London, UCL School of Pharmacy<br>Hayward, Andrew; University College London, UCL Institute of Epidemiology and Health Care; University College London, UCL Institute of Health Informatics
**Primary Subject Heading**:	Infectious diseases
Secondary Subject Heading:	Research methods, Public health
Keywords:	Tuberculosis < INFECTIOUS DISEASES, systematic review, treatment adherence

SCHOLARONE™ Manuscripts

only

Determinants of non-adherence to treatment for tuberculosis in high- and middle-income settings: a systematic review protocol

Fatima B. Wurie MSc*[1,2], Vanessa Cooper PhD[2], Robert Horne PhD[2], Andrew C. Hayward MD[1,3]

[1]Institute of Health Informatics, University College London, UK

[2]Centre for Behavioural Medicine, School of Pharmacy, University College London, UK

[3]Institute of Epidemiology and Health Care, University College London, UK

***Corresponding author email address: f.wurie@ucl.ac.uk**

For peer review only

For peer review only - http://bmjopen.bmj.com/site/about/guidelines.xhtml

Abstract

Introduction Treatment for tuberculosis is highly effective if taken according to prescribed schedules. However, many people have difficulty adhering to treatment which can lead to poorer clinical outcomes, the development of drug resistance, increased duration of infectivity and consequent onward transmission of infection. A range of approaches are available to support adherence but in order to target these effectively a better understanding of the predictors of poor adherence is needed. This review aims to highlight the personal, socio-cultural and structural factors that may lead to poor adherence in high- and middle-income settings.

Methods and analysis Six electronic databases: Medline, EMBASE, CINAHL, PsychInfo, The Cochrane Library, Scopus and Web of Science will be searched for relevant articles using a pre-specified search strategy. Observational studies will be targeted to explore factors that influence adherence to treatment in individuals diagnosed with tuberculosis. Screening title and abstract followed by full-text screening and critical appraisal will be conducted by two researchers. Data will be extracted using the Population, Exposure, Comparator, Outcomes, Study characteristics (PECOS) framework. For cross-study assessment of strength of evidence for particular risk factors affecting adherence we will use the Grading of Recommendations, Assessment, Development and Evaluation (GRADE) tool modified for Prognostic studies. A narrative synthesis of the studies will be compiled. A meta-analysis will be considered if there are sufficient numbers of studies that are homogenous in study design, population and outcomes.

Dissemination A draft conceptual framework will be identified that a) identifies key barriers to adherence at each contextual level (e.g. personal, socio-cultural, health systems) and b) maps the relationships, pathways, and mechanisms of effect between these factors and adherence outcomes for people with TB. The draft conceptual framework will guide targeting of adherence interventions and further research.

PROSPERO registration number: CRD42017061049

[287 words]

Strengths and limitations of this study

- Systematic review and meta-analysis of personal, socio-cultural and structural risk factors which predict poor adherence
- Will offer highest level of quantitative evidence to guide targeting of adherence interventions
- Wide variety of direct and indirect measures of adherence may hamper collation of outcomes.

For peer review only - http://bmjopen.bmj.com/site/about/guidelines.xhtml

For peer review only

Background

Rationale

Internationally, adherence to treatment for tuberculosis is recognised as a key tenet of the TB Elimination Framework within low-incidence countries[i] yet little is known of the personal, social and cultural factors that drive non-adherence across different groups in these settings. Poor adherence is cited as the primary reason for sub-optimal clinical benefit[ii] and leads to poorer clinical outcomes, the development of drug resistance, increased duration of infectivity and consequent onward transmission of infection. DOTS (directly-observed therapy, short-course) is the international standard for TB control. The standard 'short-course' regimen to treat drug-sensitive TB is six months. For those diagnosed with multi-drug resistant TB (MDR-TB) this regimen increases to nine to 20 months and adverse effects are more common under these regimens. Despite the plethora of effective TB regimens that exist the treatment completion rates are low and vary across different groups and may hamper international efforts to control TB. The long duration of any current effective treatment regimen for TB can make it difficult for patients to take their drugs as prescribed. For example, in 2014 in England 73.3% of short-course treated patients completed treatment under these circumstances by six to eight months and 84.5% within a year[iii]. The outcome is worse for MDR or rifampicin resistant TB cases notified in 2013, where only 57.8% had done so by 24 months. These findings indicate that the treatment and clinical outcomes are considerably poor. .

Some studies have used quantitative methods to evaluate these approaches for TB, many of which been conducted in resource-poor settings. Other qualitative studies[iv] in resource-poor settings have highlighted that poverty and gender discrimination, the social context, health service factors and personal factors interact affecting adherence to treatment.

The NICE Medicines Adherence Guidelines[v] recommended that support should be tailored to meet the needs of the individual by addressing both the perceptual factors (e.g. beliefs about the illness and treatment)[vi] and practical factors (e.g. capability, resources and opportunity) influencing the motivation and ability to start and continue with treatment[vii]. This can be summarised as a Perceptions and Practicalities Approach (PAPA)[viii]. The extent to which these factors exist or are important in predicting non-adherence to TB treatment in resource-rich settings are yet to be explored.

For peer review only - http://bmjopen.bmj.com/site/about/guidelines.xhtml

Objectives

This systematic review aims to identify the personal, socio-cultural and structural factors associated with poor adherence to treatment for TB in high- and middle-income settings. A better understanding of these factors will better inform development of interventions to strengthen a patient-centred approach for the delivery of TB programmes and services.

For peer review only

Review questions:

What are the determinants of non-adherence to treatment in patients with tuberculosis in high- and middle-income settings?

Methods

Eligibility criteria

Study design/characteristics:

Inclusion criteria

Empirical studies employing prospective, longitudinal, cross-sectional or retrospective designs. Randomised and non-randomised prospective comparative studies of interventions will be included if any predictors were found to have increased adherence and continuation of TB treatment.

The active TB condition has to be defined in the study using a clinical diagnosis. For studies presenting treatment completion rates, a definition for completion will have to be provided.

Studies conducted in high- (a GNI per capita of $12,476 or more) and upper-middle-income settings (a GNI per capita between $4,036 and $12,475 as calculated using the World Bank Atlas method for the current 2017 fiscal year will be included in the review.

For peer review only - http://bmjopen.bmj.com/site/about/guidelines.xhtml

Exclusion criteria

Studies conducted in resource-limited settings (GNI per capita of $1,025 or less in 2015 as calculated by the World Bank Atlas methods for the current 2017 fiscal year will be excluded because reviews in this area have focused already on low- and low to middle-income settings[ix]

No restrictions on age, gender or ethnicity of participants.

Participants: Individuals clinically diagnosed with active TB

Exposure: The primary exposures of interest are the risk factors that may influence adherence. Thus, studies reporting on patient demographics, knowledge and attitudes, characteristics of TB disease, social characteristics of patients, service-related factors and comorbidities will be included in the review.

Outcomes: Studies will be included in the review if the primary outcome is non-adherence. Non-adherence will be determined by self-reporting through attendance at follow-up appointments, collecting prescriptions from clinics, pill counts and pharmacy reports, electronic devices (medication events monitoring systems (MEMS) caps), urine inspection, testing for drug levels and directly-observed therapy (DOT) attendance or video-observed therapy (VOT) sessions. Studies that report outcomes such as non-completion of treatment and/or lost to follow-up and/or treatment refusal will also be included.

Information sources: Electronic databases: Medline, EMBASE, CINAHL, PsychInfo, The Cochrane Library, Scopus and Web of Science will be searched. The reference lists of relevant systematic reviews will be screened to find primary articles.

Search strategy: We will carry out medical subject heading (MeSH) terms and keyword searches for tuberculosis, treatment adherence and compliance. We will seek expert consultation from a librarian on our draft search strategy, which will combine MeSH and free text terms (including term explosion) for tuberculosis. No filters for study type will be applied for TB studies. We will remove editorials, news items and letters. Articles published in English will be included. No limits on year of publication will be applied. We have included a draft strategy for Medline in a supplementary file.

For peer review only - http://bmjopen.bmj.com/site/about/guidelines.xhtml

The list of proposed search terms will be reviewed by all authors and any necessary adjustments made prior to running the search. We will review the reference lists of eligible articles and relevant reviews to identify additional papers not indexed in the databases searched.

Data management: Output from the searched databases will be exported into Endnote version. X.7.7.1. and duplicate records removed electronically. Screening and extraction will occur in a Microsoft Access database to ensure that all retrieved references are fully tracked

Selection process: For the initial screening stage, two authors (FW and VC) will select articles by screening the title and abstract to assess whether they fulfil the study eligibility criteria. Two researchers (FW and VC) will conduct abstract selection and critical appraisal of the full-text articles. To reduce the risk of missing potentially relevant studies, researchers will adopt a more lenient approach at the first level of screening. Both researchers will obtain full-text articles for studies that meet the review inclusion criteria. Reasons for rejection of articles during both the initial screening and at the full-text screening process will be noted and any discrepancies will be discussed by FW and VC and consultation with AH and RH will be done if necessary.

Data extraction: We will use the Population, Exposure, Comparator, Outcomes, Study characteristics (PECOS) framework to systematise data extraction. Data will be extracted using a standardised template containing information on each of the following 5 domains:

Population: characteristics of the study population (clinically diagnosed with active tuberculosis and recommended for treatment), recruitment and sampling methods, inclusion/exclusion criteria);

Exposure: any risk factors that may influence adherence. Including patient demographics (age and sex distribution, ethnicity), BCG vaccination status, knowledge and attitudes of TB, characteristics of TB (including drug-resistant strain status, co-infection status), social characteristics of patients, service-related factors, interventions and comorbidities, number of exposed subjects, any exclusions;

Comparators: identification and definition of unexposed subjects, any exclusions;

Outcomes: definition and identification of adherence levels for TB, non-completion of concomitant treatment, loss to follow-up, treatment refusal, number of subjects, any exclusions, length of follow-up;

For peer review only - http://bmjopen.bmj.com/site/about/guidelines.xhtml

Study characteristics: authors, publication year, setting/source of participants, design, period of study, length if follow-up time (if relevant), aims and objectives. Unadjusted and fully-adjusted effect estimates for the association between TB and adherence will be recorded. Details of confounders measured and adjusted will also be noted. Any results from additional stratified analyses will also be recorded.

We will consider contacting corresponding authors to obtain any missing information using a standardised email template.

Risk of bias quality assessment (in individual studies): Risk of bias domains will be used from the Cochrane Collaboration for specific observational study designs. Assessment of risk of bias of individual studies and outcomes will be conducted by two reviewers independently and will subsequently discuss amongst all authors for arbitration. For cross-study assessment of strength of evidence for particular risk factors affecting adherence the GRADE tool modified for Prognostic studies[x] will be used. Specifically, differential outcome measurement in exposed and unexposed cohort populations, incomplete follow-up, failure to control for confounding, difference in measurement of exposure and selection of exposed and unexposed in cohort studies from different populations will be examined. We will examine each outcome for risks of bias, inconsistency, indirectness, imprecision, publication bias and any additional domains deemed appropriate. We will prioritise direct objective measures of adherence, which are less prone to reporting bias.

Strategy for data synthesis: A high proportion of studies either reporting adherence during the first two months (initiation phase of treatment) or throughout treatment and use of wide variety of instruments are anticipated. Adherence measures at 2- and 6-month time points will be measured separately. Use of different instruments will be reported separately. Any consistency of identified risk factors across instruments and for the different time periods will be reported separately. Further analyses of MDRTB patients as well as considering MDRTB as a risk factor for adherence outcomes will be conducted. Sub-analyses to assess whether treatment regimens are predicators of non-adherence will be performed. A narrative synthesis will be compiled of the studies, including a consideration of the socio-economic context in which included interventions were implemented and other critical factors, such as the drug resistance profiles of the study population. The evidence tables will be arranged and divided according to the different treatment durations and regimens for treatment for TB.

For peer review only - http://bmjopen.bmj.com/site/about/guidelines.xhtml

Meta-analysis: If there are sufficient numbers of studies that are homogenous in study design, population and outcomes we will obtain a pooled effect estimate. The choice of fixed or random effects model will be guided by the level of statistical heterogeneity (assessed using the I^2 statistic). A p value for I^2 of less than 0·05 will indicate that heterogeneity among the group of studies being analysed was significant. If the I^2 statistic is greater than 50% (with $p<0·05$) for each treatment outcome, a random-effects analysis, incorporating the impact of both chance and heterogeneity among study populations and study design, will be chosen over the fixed-effects alternative, which assumes that differences among study outcomes are due entirely to chance. We will use STATA to conduct our meta-analysis.

To assess study quality, we will use the Grading of Recommendations, Assessment, Development and Evaluation (GRADE) approach, in which quality of the body of evidence is examined for each outcome rather than by individual study. We will use GRADEpro software to create summary of findings tables.

Ethics and dissemination: Ethical review is not required as this study is a systematic review. It is our intention to submit the findings of this review to a peer-reviewed journal and to present at national and international symposia. Based on the results of the systematic review, we will develop a draft conceptual framework that a) identifies key barriers to adherence at each contextual level (e.g. personal, socio-cultural, health systems) and b) maps the relationships, pathways, and mechanisms of effect between these factors and adherence outcomes for TB patients on treatment. The draft conceptual framework will guide research questions and formative primary research to understand the factors that influence patterns of irregular and non-adherence.

[This protocol has been prepared using the Preferred Reporting Items for Systematic Reviews and meta-Analyses Protocols (PRISMA-P) guidelines[xi]]

For peer review only - http://bmjopen.bmj.com/site/about/guidelines.xhtml

Contributorship statement

ACH and FW conceived the idea, planned and designed the study protocol. FW planned the statistical analysis and data extraction. RH and VC provided critical insights. All authors have approved and contributed to the final written manuscript.

Competing interests

None

Funding statement

No funding to report for this submission

Data sharing statement

This study is a systematic review protocol and no unpublished data has arisen from this proposed work.

For peer review only

For peer review only - http://bmjopen.bmj.com/site/about/guidelines.xhtml

References

[i] World Health Organisation. TB Elimination Framework. Geneva. 2014
[ii] World Health Organisation. Adherence to long term therapies. Geneva; Jan. 2003 http://www.who.int/chp/knowledge/publications/adherence_report/en/
[iii] Public Health England. Tuberculosis in England: 2016. Sept. 2016. https://www.gov.uk/government/publications/tuberculosis-in-england-annual-report
[iv] Munro SA, Lewin SA, Smith HJ, Engel ME, Fretheim A, Volmink J. Patient Adherence to Tuberculosis Treatment: A Systematic Review of Qualitative Research. PLoS Med. 2007 Jul 24;4(7):e238
[v] National Institute for Health and Clinical Excellence. Medicines adherence: involving patients in decisions about prescribed medicines and supporting adherence. NICE guidelines [CG76]. Jan 2009.
[vi] Horne R, Weinman J, Barber N, Elliott R, Morgan M. Concordance, adherence and compliance in medicine taking. Dec 2005. http://www.nets.nihr.ac.uk/__data/assets/pdf_file/0009/64494/FR-08-1412-076.pdf
[vii] Chang S-H, Cataldo JK. A systematic review of global cultural variations in knowledge, attitudes and health responses to tuberculosis stigma. Int J Tuberc Lung Dis Off J Int Union Tuberc Lung Dis. 2014;18:168–73.
[viii] Horne R, Weinman J, Barber N, Elliott R, Morgan M. Concordance, adherence and compliance in medicine taking. Dec 2005. http://www.nets.nihr.ac.uk/__data/assets/pdf_file/0009/64494/FR-08-1412-076.pdf
[ix] Munro SA, Lewin SA, Smith HJ, Engel ME, Fretheim A, Volmink J. Patient Adherence to Tuberculosis Treatment: A Systematic Review of Qualitative Research. PLoS Med. 2007 Jul 24;4(7):e238
[x] Iorio *A et al* Use of GRADE for assessment of evidence about prognosis: rating confidence in estimates of event rates in broad categories of patients *BMJ 2015;350:h870*
[xi] Shamseer L, Moher D, Clarke M, et al. Preferred reporting items for systematic review and meta-analysis protocols (PRISMA-P) 2015: elaboration and explanation. BMJ 2015;349:g7647. doi:10.1136/bmj.g7647

For peer review only

For peer review only - http://bmjopen.bmj.com/site/about/guidelines.xhtml

Determinants of non-adherence to treatment for tuberculosis in high- and middle-income settings: a systematic review protocol

Search strategy for Medline

1. (tuberculosis.mp. or exp tuberculosis, human/ or TB/) not latent.mp. [mp=title, abstract, original title, name of substance word, subject heading word, keyword heading word, protocol supplementary concept word, rare disease supplementary concept word, unique identifier, synonyms]

2. exp patient acceptance of health care/

3. adher*.mp.

4. ((non or "not") adj3 initiat*).mp.

5. ((non or "not") adj3 complet*).mp.

6. ((non or "not") adj3 complian*).mp.

7. lost to follow up.mp.

8. nonadher*.mp.

9. ((non or "not") adj3 concord*).mp.

10. (DOTS or directly observed therap* or treat*).mp.

11. 2 or 3 or 4 or 5 or 6 or 7 or 8 or 9 or 10

12. 1 and 11

For peer review only

PRISMA-P (Preferred Reporting Items for Systematic review and Meta-Analysis Protocols) 2015 checklist: recommended items to address in a systematic review protocol*

Section and topic	Item No	Checklist item
**ADMINISTRATIVE INFORMATION**		
Title:		
Identification	1a	Identify the report as a protocol of a systematic review
Update	1b	If the protocol is for an update of a previous systematic review, identify as such
Registration	2	If registered, provide the name of the registry (such as PROSPERO) and registration number
Authors:		
Contact	3a	Provide name, institutional affiliation, e-mail address of all protocol authors; provide physical mailing address of corresponding author
Contributions	3b	Describe contributions of protocol authors and identify the guarantor of the review
Amendments	4	If the protocol represents an amendment of a previously completed or published protocol, identify as such and list changes; otherwise, state plan for documenting important protocol amendments
Support:		
Sources	5a	Indicate sources of financial or other support for the review
Sponsor	5b	Provide name for the review funder and/or sponsor
Role of sponsor or funder	5c	Describe roles of funder(s), sponsor(s), and/or institution(s), if any, in developing the protocol
**INTRODUCTION**		
Rationale	6	Describe the rationale for the review in the context of what is already known
Objectives	7	Provide an explicit statement of the question(s) the review will address with reference to participants, interventions, comparators, and outcomes (PICO)
**METHODS**		
Eligibility criteria	8	Specify the study characteristics (such as PICO, study design, setting, time frame) and report characteristics (such as years considered, language, publication status) to be used as criteria for eligibility for the review
Information sources	9	Describe all intended information sources (such as electronic databases, contact with study authors, trial registers or other grey literature sources) with planned dates of coverage
Search strategy	10	Present draft of search strategy to be used for at least one electronic database, including planned limits, such that it could be repeated
Study records:		
Data management	11a	Describe the mechanism(s) that will be used to manage records and data throughout the review

Selection process	11b	State the process that will be used for selecting studies (such as two independent reviewers) through each phase of the review (that is, screening, eligibility and inclusion in meta-analysis)
Data collection process	11c	Describe planned method of extracting data from reports (such as piloting forms, done independently, in duplicate), any processes for obtaining and confirming data from investigators
Data items	12	List and define all variables for which data will be sought (such as PICO items, funding sources), any pre-planned data assumptions and simplifications
Outcomes and prioritization	13	List and define all outcomes for which data will be sought, including prioritization of main and additional outcomes, with rationale
Risk of bias in individual studies	14	Describe anticipated methods for assessing risk of bias of individual studies, including whether this will be done at the outcome or study level, or both; state how this information will be used in data synthesis
Data synthesis	15a	Describe criteria under which study data will be quantitatively synthesised
	15b	If data are appropriate for quantitative synthesis, describe planned summary measures, methods of handling data and methods of combining data from studies, including any planned exploration of consistency (such as I^2, Kendall's τ)
	15c	Describe any proposed additional analyses (such as sensitivity or subgroup analyses, meta-regression)
	15d	If quantitative synthesis is not appropriate, describe the type of summary planned
Meta-bias(es)	16	Specify any planned assessment of meta-bias(es) (such as publication bias across studies, selective reporting within studies)
Confidence in cumulative evidence	17	Describe how the strength of the body of evidence will be assessed (such as GRADE)

*** It is strongly recommended that this checklist be read in conjunction with the PRISMA-P Explanation and Elaboration (cite when available) for important clarification on the items. Amendments to a review protocol should be tracked and dated. The copyright for PRISMA-P (including checklist) is held by the PRISMA-P Group and is distributed under a Creative Commons Attribution Licence 4.0.**

From: Shamseer L, Moher D, Clarke M, Ghersi D, Liberati A, Petticrew M, Shekelle P, Stewart L, PRISMA-P Group. Preferred reporting items for systematic review and meta-analysis protocols (PRISMA-P) 2015: elaboration and explanation. BMJ. 2015 Jan 2;349(jan02 1):g7647.
